# The Prognosis of ART Is Not Altered in Cystic Fibrosis Women: A Case-Report Study

**DOI:** 10.3389/fendo.2022.773753

**Published:** 2022-02-21

**Authors:** Inès Braham, Adeline Morisot, Samir Boukaïdi, Marie Perceval, Isabelle Durieu, Christine Rousset-Jablonski, Sylvie Hieronimus, Sylvie Leroy, Nicolas Chevalier

**Affiliations:** ^1^ Université Côte d’Azur, CHU de Nice, Département d’Endocrinologie-Diabétologie et Reproduction, Nice, France; ^2^ Université Côte d’Azur, CHU de Nice, Département de Santé Publique, Nice, France; ^3^ Université Côte d’Azur, CHU de Nice, Centre d’aide médicale à la procréation, Nice, France; ^4^ Hospices Civils de Lyon, CHU de Lyon, Centre de Ressources et de Compétences de la Mucoviscidose, Lyon, France; ^5^ Hospices Civils de Lyon, CHU de Lyon, Service de gynécologie-obstétrique, Lyon, France; ^6^ Université Côte d’Azur, CHU de Nice, Centre de Ressources et de Compétences de la Mucoviscidose, Nice, France; ^7^ Université Côte d’Azur, Inserm U1065, C3M, Nice, France

**Keywords:** cystic fibrosis, female, infertility, assisted reproductive techniques, pregnancy, pregnancy outcome

## Abstract

**Research Question:**

Unlike in men, a very limited number of studies were focused on the specificity of ART management of cystic fibrosis (CF) in women. The purpose of this study was to determine the causes of infertility in patients, the appropriate ART treatment, and their prognosis in terms of pregnancy.

**Design:**

We conducted a multicentre analytical case-control study including CF women who were age-matched to non-CF women. We reported the causes of infertility, the ART management type and pregnancy outcomes.

**Results:**

17 cases were compared to 34 controls. There was no significant difference between the groups concerning cause infertility. There was a non-statistically significant trend with a lower antral follicle count in CF compared to controls (19.5 versus 26.8, p=0.08). IUI seemed to be as successful as IVF/ICSI in CF as opposed to controls where the IVF/ICSI was the most effective (in CF group for HCG >100 UI/L: 38.8% vs. 36.8%, p=0.4175). There were more embryos obtained in CF than in controls (3.1 versus 1.6, p=0.02). The number of oocytes and embryos obtained and pregnancy outcomes remained similar between DF508 homozygous group and others CFTR mutations group. The results of ART procedures and pregnancy evolution were not influenced by FEV1.

**Conclusion:**

In absence of any other pathology, IUI may be first option for CF women. If insemination fails, IVF with a low dose of gonadotropins may be more appropriate to prevent the risk of hyperstimulation syndrome. FEV1 and genetic do not seem to be contributing factors in the prognosis of ART.

## Highlights

There is a significant part of cervical origin in cystic fibrosis subfertility. Intrauterine insemination may be proposed as a first option. If insemination fails, IVF with low doses of gonadotropins may be proposed to avoid hyperstimulation syndrome. Only one embryo should be transferred because of the increased risk of prematurity.

## Introduction

Cystic fibrosis (CF) is a common severe genetic disorder in Caucasian population. In Europe and in North America, CF affects 1 in 2500 births (1). In France in 2018, 7180 patients were identified by the national register centre.

CF is caused by mutations in the cystic fibrosis transmembrane conductance regulator (CFTR, also named ABCC7) gene leading to abnormal mucus in the lung, pancreas, gastro-intestinal tract and reproductive tract (2). In 2012, the first CFTR modulator called ivacaftor was introduced to the market. This new therapy potentiates the probability of opening (or regulation) of the CFTR channel at the cell surface (3), while tezacaftor and elexaftor, later commercialised, facilitate the maturation and the transfer of the CFTR protein to the cell surface (4). In 2020, the triple combination therapy constituted of ivacaftor, tezacaftor and elexaftor was approved (4). Associated with the optimization of multidisciplinary management, this new treatment has drastically reduced the mortality of CF patients (12.4‰ in 2006 to 7.9‰ in 2018 in France) and improved the life expectancy (from 7-year-old in 1965 to 47-year-old) (5).

As a result of increased life expectancy, the question of parenthood for these patients has become now more relevant than before. The fertility of CF males has been widely studied. 97% of the CF males experimented obstructive azoospermia due to congenital bilateral absence of vas deferens in addition to atrophic or absent seminal vesicles (6). Several studies suggested that the atresia of vas deferens resulted from the obstruction of the lumen due to abnormal mucus (6). However, CFTR protein seems to affect the different signaling pathways of spermatogenesis and spermiogenesis. Indeed, CFTR is involved in tight junctions’ formation of the seminiferous tubules which are essential to the haemato-testis barrier. CFTR can also affect transcription factors such as CREB which is essential to sperm nuclear integrity (7). Moreover, other studies (8, 9) have demonstrated that the CFTR protein was involved in the production of bicarbonate molecule in female genital tract which is essential to capacitation.

Regarding CF in women, recent studies (10, 11) have estimated the rate of hypofertility to be around 35-40% while it was only 10% in the overall European population. The primary hypothesis of this subfertility was related to cervical mucus being thicker and as a result acting as a barrier to the passage of the sperm (12). Other studies suggested that it could be also related to abnormal uterine secretions impeding the flow of sperm in the female genital tract (13). Ovarian failure was also suspected as animal studies have shown that the CFTR protein was expressed in the hypothalamus (14) and in the ovaries (15). Thus, the mechanisms of subfertility among CF females are not fully understood despite several identified contributing factors: hypothalamic factor, anovulation, ovarian failure, cervical and tubal causes, with the CFTR protein as a common denominator. Furthermore, when those patients must undergo assisted reproductive technologies (ART) in order to obtain pregnancy, the prognosis of ART is not well-known. In this context, we conducted a retrospective multicentre study to analyze fertility parameters, ART procedures and pregnancy outcomes among CF women compared to non-CF women.

## Materials and Methods

### Population Recruitment

We conducted a retrospective multicentre analytical case-control study including the CF women between 18 and 42 yrs. who were referred to CF reference centres (CRCM) of Nice University Hospital and Hospices Civils of Lyon between 01/01/2002 and 04/30/2019. These patients were concomitantly treated in ART units in Lyon or Nice. Patients with a significant amount of data missing (n=16) or spontaneously pregnant prior to ART (n=1) were not included in the study. The control group included randomly selected non-CF women treated in Nice ART unit. Controls were matched to patients according to their age at the time of ART treatment and we chose two controls for each CF-patient. All patient husbands underwent a genetic testing before ART procedures and were negative for the CFTR mutations.

Our study was approved by French National Commission on Information Technology and Liberties (R04-001 and MR 1415150219). All participants gave their informed consent and all the data were anonymized before being analyzed.

### Outcomes

The main objective of our study was to determine the causes of infertility in CF women, between:

-ovulation disorders, classified according to the WHO standard (16) as type I (central origin), type IIa (idiopathic eugonadotropic ovulation disorder), type II b (polycystic ovary syndrome), type III (ovarian failure);
-tubal cause defined by the absence or the obstruction of either tubes which can be identified by hysterosalpingography or by laparoscopy;
-cervical cause, identified through a negative postcoital test confirming the absence of sperm in the cervical mucus;
-uterine cause, due to a malformation (such as uterine septum, unicornuate uterus, pseudo-unicornuate, hypoplastic T-shaped uterus) or endometriosis, regardless of the stage;
-male cause in case of any abnormality of the spermogram or spermocytogram;
-mixed cause defined by the presence of two or more types of infertility factors;
-or idiopathic cause.

Fertility parameters such as antral follicle count (AFC), FSH, hysterosalpingography (conducted around day 10 of the cycle), postcoital test, cycle regularity or age of menarche were collected. The AFC and FSH (in IU/L) were performed between the second and the fourth day of the cycle. The number of the antral follicles was assessed by an endovaginal 5-9 MHz probe counting all the follicles with a diameter between 2 and 10 mm. AMH analysis were not considered in our study as the assay kits were different between the two centres and changed twice in Nice during our study.

Secondary objectives included the type of ART procedures (ovulation induction [OI], artificial intrauterine insemination [IUI], *in vitro* fertilization [IVF] or intracytoplasmic sperm injection [ICSI], artificial insemination with sperm donor or IVF with donated oocytes) and the pregnancy rate. For this last parameter, we considered biological pregnancy as women with positive betaHCG under 100 UI/L at day 14 after insemination or fertilization; viable pregnancy as women with positive betaHCG over 100 UI/L at day 14 with a doubling kinetics during the following week. These pregnancies were confirmed as clinical pregnancy by ultrasonographic visualization of one or more gestational sacs. We also considered the number of ectopic pregnancies, pregnancy loss and finally evaluated the number of live births in each group.

The number of oocytes, the number of embryos obtained for each attempt at IVF in the 2 groups, the occurrence of hyperstimulation and the number of attempts before pregnancy and live birth were also collected for each participant of the study.

### Statistical Analysis

Results were presented as mean and deviation-type for quantitative variables, and median and interquartile range for the other variables.

For the comparison between cases and controls, the Student test (also referred as Mann-Whitney nonparametric test) or the Chi-square test (also referred as Fisher test) were used, depending on the nature of the variables considered. The value of the error of first test was set at 5%.

## Results

### Characteristics of the Population

Among 217 CF women treated in Lyon Sud and Nice’s CRCM, 34 were concomitantly treated in ART units but only 17 women could be included in our study and matched to 34 controls according to their age. The characteristics of CF patients and their controls are detailed in **Table 1**. Case and controls were not different except for weight and body mass index which were significantly lower in CF women than in controls (p=0.004 and 0.0018, respectively). No patient underwent pulmonary transplant before ART treatment. Only one patient took CFTR modulators before ART treatment. When we compared the patients according to their genetic status (DF508 homozygous mutation [n=8] or other mutations [n=9]), we observed that more DF508 patients experimented history of pulmonary surgery (p=0.02). Furthermore, homozygous DF508 patients seemed to be younger and to exhibit more frequently an exocrine pancreatic insufficiency (**Table 1**).

**Table 1:** Characteristics of the population and comparison between the genetic status of the cases.

	Controls (n= 34)	CF (n= 17)	p (Controls vs CF)	DF508 homozygous (n= 8)	Other mutations (n= 9)	P (DF508 vs other mutations)
**Age (yrs)**	29.3 (± 4.1)	29.3 (± 4.2)	NS	27.1 (± 3.1)	31 (± 5)	NS
**Weight (kg)**	61.7 (± 11.7)	52.7 (± 5.9)	0.004	52.6 (± 6.1)	52.8 (± 6)	NS
**BMI (kg/m^2^)**	23.6 (± 3.8)	20.3 (± 1.8)	0.0018	20.4 (± 2.2)	20.2 (± 1.6)	NS
**History of spontaneous pregnancy**	0.3 (± 0.7)	0.2 (± 0.4)	NS	0.1 (± 0.4)	0.2 (± 0.4)	NS
**History of ART pregnancy**	0.1 (± 0.4)	0.1 (± 0.3)	NS	0.1 (± 0.4)	0.1 (± 0.3)	NS
**Pulmonary Surgery**		0.2 (± 0.4)		0.5 (± 0.5)	0 (± 0)	0.0221
**CFTR modulators**		0.1 (± 0.2)		0.1 (± 0.4)	0 (± 0)	NS
**FEV1 (%)**		63.2 (± 18)		67.1 (± 24.7)	59.4 (± 7)	NS
**Diabetes**		0.5 (± 0.5)		0.5 (± 0.5)	0.4 (± 0.5)	NS
**Exocrine insufficiency**		0.8 (± 0.4)		1 (± 0)	0.7 (± 0.5)	NS
**Pseudomonas aeruginosa colonization**		0.9 (± 0.3)		1 (± 0)	0.8 (± 0.4)	NS

Results are expressed as mean ± SD.

NS, non-significant; CFTR, cystic fibrosis transmembrane conductance regulator; ART, assisted reproductive technology; FEV1, forced expiratory volume in 1 second.

### Fertility Parameters

Fertility parameters of CF women and controls are detailed in **Table 2**. There was no significant difference between the two groups concerning infertility’s etiology. However, no conclusion can be drawn in respect of cervix cause due to the lack of data in the control group. There is no difference concerning ovulation disorders type III between CF women and control (23.5% versus 8.8%, p=0.5345). Mean AFC were similar in both groups (19.5 ± 10.8 in CF women vs 26.8 ± 13.5 in controls; p = 0,0884) as FSH levels, age at menarche and cycle duration (**Table S1**).

**Table 2:** Infertility’s etiologies of the population.

	CF (n = 17)	Controls (n = 34)	P
**Tubal cause**	4 (23.5%)	4 (12.9%)	NS
**No tubal cause**	13 (76.5%)	27 (87.1%)	
*Missing data*	0	3	
**Cervix cause**	9 (100%)	7 (77.8%)	NS
**No cervix cause**	0 (0%)	2 (22.2%)	
*Missing data*	8	25	
**Ovulation disorders type I**	0 (0%)	1 (2.9%)	NS
**Ovulation disorders type II a,b**	5 (29.4%)	11 (32.4%)	
**Ovulation disorders type III**	4 (23.5%)	3 (8.8%)	
**No ovulation disorders**	8 (47.1%)	19 (55.9%)	
**Uterine disorders- endometriosis**	2 (11.8%)	5 (14.7%)	NS
**No uterine disorders- endometriosis**	15 (88.2%)	29 (85.3%)	
**Male infertility**	8 (47.1%)	19 (55.9%)	NS
**No male infertility**	9 (52.9%)	15 (44.1%)	
**Idiopathic**	1 (5.9%)	2 (5.9%)	NS
**No idiopathic**	16 (94.1%)	32 (94.1%)	
**Mixt**	9 (52.9%)	14 (41.2%)	NS
**No mixt**	8 (47.1%)	20 (58.8%)	

Results are expressed as absolute values and as percentages in the group (column analysis).

NS, non-significant.

### ART Procedures and Outcomes

A total of 221 ART procedures were performed in CF women (n = 66) and controls (n = 155). IUI were more practiced in CF women than in control group (39.4% [n=26/66] vs. 28.4% [n=44/155]; p=0.0425), while IVF/ICSI was the most frequent ART procedure in control group (41.3% [n=64/155] vs. 27.3% [n=18/66]; p=0.0425). Ovulation induction and fresh embryo transfer were similarly performed in both groups (respectively 3/66 [4.5%] and 19/66 [28.8%] in CF women, and 17/155 [11%] and 30/155 [19.4%] in control group).

ART outcomes are detailed in **Table 3**. There is no significant difference between the two groups concerning HCG outcome, ovarian hyperstimulation rate, number of oocytes collected and number of embryos transferred. However, more embryos were obtained in CF women than in the control group (3.1 vs 1.6; p=0.02) and the number of transferred embryos before HCG>100UI/L was significantly lower in CF women than in control group (1.0 ± 0.0 vs. 2.5 ± 1.8; p=0.0295). Nevertheless, the number of pregnancy loss and live births were similar in both groups (**Table 3**).

**Table 3:** ART outcomes and pregnancy outcomes.

	CF (n = 66)	Controls (n = 155)	p
**Ovarian hyperstimulation**	3 (4.5%)	10 (6.5%)	NS
**No ovarian hyperstimulation**	63 (95.5%)	145 (93.5%)	
**HCG >100 UI/L**	19 (28.8%)	35 (22.6%)	NS
**HCG <100 UI/L**	3 (4.5%)	4 (2.6%)	
**HCG negative**	44 (66.7%)	116 (74.8%)	
**Clinical pregnancy**	17 (25.8%)	34 (21.9%)	NS
**No clinical pregnancy**	49 (74.2%)	121 (78.1%)	
**Pregnancy loss, ectopic pregnancy**	8 (12.1%)	12 (7.7%)	NS
**No pregnancy loss ectopic pregnancy**	58 (87.9%)	143 (92.3%)	
**Number of oocytes collected**	10.1 (± 5.9)	9.1 (± 5.3)	NS
**Number of embryos obtained**	3.1 (± 2.4)	1.6 (± 1.5)	0.0246
**Abandon in IVF**	1 (6.7%)	5 (7.8%)	NS
**No abandon in IVF**	14 (93.3%)	59 (92.2%)	
*Missing data*	3	0	
**No embryo transferred in IVF**	8 (53.3%)	22 (34.4%)	NS
**Transfer**	7 (46.7%)	42 (65.6%)	
*Missing data*	3	0	
**Number of embryos transferred (IVF and FET)**	0.8 (± 0.4)	0.9 (± 0.7)	NS
**Switch IVF to IUI**	0 (0%)	1 (1.6%)	NS
**No switch**	15 (100%)	63 (98.4%)	
*Missing data*	3	0	
**Live births**	10 (15.2%)	23 (14.8%)	NS
**No live births**	56 (84.8%)	132 (85.2%)	

Results are expressed as mean ± SD.

Results are expressed as absolute values and as percentages in the group (column analysis).

NS, non-significant; IUI, intrauterine insemination; IVF, in vitro fertilisation; FET, frozen embryo transfer.

Ovarian hyperstimulation was significantly associated with IVF/ICSI procedures in both groups (p=0.0432 in CF women and 0.0018 in controls) (**Tables S2**, **S3**). There was no difference between the different procedures regarding the number of pregnancy loss and live births in CF women, especially between IUI and IVF/ICSI (**Table S2**). At the opposite, in control group, IVF/ICSI significantly led to more issues with HCG > 100 UI/L (48.6% vs 14.3%; p=0.0011) but the number of live births were similar between IVF/ICSI and IUI subgroups in control women (respectively 56.5 and 21.7%; p=0.4507). Fresh embryo transfer was associated with an increase of pregnancy loss and ectopic pregnancies in control women (66.7% vs 0%; p=0.0004) (**Tables S2**, **S3**). Estimated live birth rate was 11,1% (52/469 cycles) for IVF/ICSI and 19.4% for IUI (13/67 cycles) in our ART center, not different than the live birth rate observed in CF women (p=0.07 and 0.43 respectively). Estimated clinical pregnancy rate was 11,3% (53/469 cycles) for IVF/ICSI, significantly lower than observed in CF women (p=0.01), and was 17.9% for IUI (12/67 cycles) in our ART center, not different than observed in CF women (p=0.59).

We compared the ART and pregnancies outcomes depending on genotypes of each participant, considering two groups: patients who were homozygous for the classical DF508 mutation (n=28) and patients with all other mutations (n=38). Results are detailed in **Table 4**. In patients who were homozygous for the classical DF508 mutation, IUI was significantly most frequently used (50.0% vs 31.6%; p=0.0498) while IVF/ICSI was most significantly used in patients exhibiting other mutations (34.2% vs 17.9%; p=0.0498). However, there was no significant difference between the two groups concerning the number of collected oocytes, the number of obtained embryos and pregnancy outcomes.

**Table 4:** ART outcomes and pregnancy outcomes according to genotype sampling.

	Other mutations (n = 38)	DF508/DF508 (n = 28)	p
**Oocytes**	9.6 (± 6.5)	11.2 (± 4.9)	NS
**Live births**	5 (13.2%)	5 (17.9%)	NS
**No live births**	33 (86.8%)	23 (82.1%)	
**Clinical pregnancy**	8 (21.0%)	9 (32.1%)	0.390 NS
**No clinical pregnancy**	30 (79.0%)	19 (67.9%)	
**HCG >100 UI/L**	9 (23.7%)	10 (35.9%)	NS
**HCG <100 UI/L**	3 (7.9%)	0 (0%)	
**HCG negative**	26 (68.4%)	18 (64.3%)	
**Number of embryos obtained**	3 (± 2.5)	3.2 (± 2.5)	NS
**IVF/ICSI**	13 (34.2%)	5 (17.9%)	0.0498
**IUI**	12 (31.6%)	14 (50%)	
**OI**	0 (0%)	3 (10.7%)	
**FET**	13 (34.2%)	6 (21.4%)	

Results are expressed as mean ± SD.

Results are expressed as absolute values and as percentages in the group (column analysis).

NS, non-significant; IUI, intrauterine insemination; IVF, in vitro fertilization; ICSI, intracytoplasmic sperm injection; OI, ovulation induction; FET, frozen embryo transfer.

We also analyzed the pregnancy outcomes depending on previous FEV1 (Forced Expiratory Volume in the first second). We observed that FEV1 was similar in women with HCG < 100 UI/L (n=48) and in women with HCG > 100 UI/L (n=18) (60.0 ± 8.6 vs 63.9 ± 16.3% respectively; p=0.7568). The same observation was performed between women with (n=10) or without live birth (n=56) (69.9 ± 21.1 vs 59.7 ± 8.3% respectively; p=0.1461).

## Discussion

A recent study (10) has shown reduced fertility in CF patients compared to the general population (37% of infertility versus 10 to 24%). Those women are usually referred to ART centres but few data are available in the literature concerning the prognosis of ART procedures in such women. In this study, we showed that the prognosis of ART in CF patients did not appear to be altered in comparison with unaffected patients.

Historically, cervical origin was the first hypothesis of this subfertility. Indeed, in 1973, Kapito et al. shown a dehydrated and thickened cervical mucus leading to abnormal cervical sperm passage in CF patients. Moreover, these authors noticed in midcycle a lower water content and the absence of sodium peak, which are usually observed just before ovulation (32). Cervical origin would seem to be a significant part of subfertility in CF patients whereas in the general population, it is commonly accepted that cervical origin accounts for less than 5% of infertilities (17). In this study, we could not prove that there were more negative postcoital tests in the CF group compared to the control group due to the small sample size. However, we showed that in the CF group IUI was just as effective as IVF/ICSI in contrast to the control group where IVF/ICSI was more effective in terms of ongoing pregnancy. To our knowledge, no case-report study has reported such data. In France in 2018, IUI represents 29.9% of ART methods against 39.1% for IVF/ICSI, which is consistent with the results of the controls in this study. These results confirm the hypothesis of a predominant cervical cause in the subfertility of CF patients and medical teams used IUI more often in order to bypass the cervical mucus.

Concerning fallopian tubes, it was found that there were 23.5% of tubal infertility in CF patients against 12.9% in controls (p=0.4285). When comparing these results with the general population, there is no difference (25%, 17). Actually, according to some authors (18), the fallopian tubes and the endometrium seem to be relatively unaffected in CF patients as a result of the low expression of CFTR protein in these structures. However, in CF patients, uterine fluid is nevertheless altered because it contains less bicarbonate (19, 20) which is essential to sperm capacitation and to oocyte fertilisation (21). These observations are contradictory to our results since we have shown that there was no more recourse to IVF than to insemination in the CF group compared to the control group (IVF/ICSI: n=18 [27.3%] and IUI: n=26 [39.4%] in the CF group versus IVF/ICSI: n=64 [41.3%] and IUI: n=44 [28.4%] in the control group, p=0.04).

Tizzano et al. did not find expression of CFTR in ovaries. On the opposite, a Canadian study (22) comparing 20 CF patients with 20 controls showed a significant decrease in AMH in the CF group. In line with this study, we reported a non-significant trend with a lower AFC in the CF group compared to the control group (19.5 versus 26.8, p=0.0884) even though we didn't report a higher proportion of ovarian failure (type III ovarian dysovulation) in our CF group.

Despite a non-significant decrease in AFC in CF patients, the oocyte response in IVF/ICSI does not seem to be lower (no difference of oocytes’ number collected between the two groups). And the yield in terms of embryos obtained is even better. This raises the question of a better oocyte quality in these patients.

In addition, we showed that the number of embryos transferred before ongoing pregnancy was lower in the CF group compared to the control group. Thus, we can assume that the uterine environment of CF patients.

Still in the hypothesis of an involvement of the CFTR gene in the functioning of the gonadotropic axis, the literature reported a more delayed puberty in CF patients: older studies (23) attributed this delay to the precarious nutritional and pulmonary condition, but with the progress of treatment and the improvement in the health status of the patients, another study (24) showed that this delay in puberty is independent of the respiratory and nutritional parameters. The mechanism of this pubertal delay is still not known but one hypothesis considers hypothalamus as a possible origin. Indeed, it has been shown that the CFTR gene is expressed in the anterior area of the hypothalamus in humans, which is itself involved in the regulation of puberty. The study by Weyler et al. reported the existence of hypothalamic neurons, called GT1-7, expressing the *CFTR* gene in rats and humans. In rats, inactivation of CFTR is responsible for a decrease in the secretion, but not the synthesis, of GnRH. The hypothesis is that this inactivation leads to a defect in the release of vesicles containing GnRH neuropeptides. These observations question the preponderance of hypogonadotropic hypogonadism in this population. We did not find any difference between CF and control group in our cohort.

The use of ART in CF patients is not without risk. Indeed, the risk of ovarian hyperstimulation or the occurrence of a multiple pregnancy could be life-threatening for the patients. Studies (25) have shown the deleterious effect of estrogens on the disease. It can therefore be assumed that hyperestrogenism induced by IVF or ICSI procedures could aggravate the disease. If IVF can impact on lung status, we question the influence of FEV1 in the prognosis in ART. We did not find any significant difference in terms of pregnancy or live birth according to previous FEV1.

In the same perspective, as the patients with homozygous DF508 mutation status have more respiratory difficulties, we wondered whether these patients had a worse prognosis in ART and a worse response in IVF. In the literature, only one study (26) has investigated the impact of *CFTR* gene mutations on the prognosis in ART. The authors reported no significant difference between the *CFTR* mutation carrier group and non-carrier patients in terms of implantation and pregnancy rates. However, the number of mature oocytes was higher but the number of embryos obtained was lower in the carrier group. In contrast to our study, the patients included were not CF patients but *CFTR* mutation carriers. In Vanwort et al. study, when interesting more specifically the type of mutation in subgroups, the authors observed an increase in the number of embryos at 2 PN and a decrease in the number of abnormally fertilised embryos in patients with the DF508 mutation.

In our study, there was no significant difference in terms of oocyte response, number of embryos obtained and live births between DF508 homozygous patients compared to patients with other mutations. However, IUI seemed to be used more often in DF508 homozygous patients compared to patients with other mutations where IVF was used more. This may be because the medical team was more reluctant to use IVF with the complications that this can cause. However, all of these results should be balanced against the fact that the patients homozygous for the DF508 mutation were younger than the other patients (trends towards the limit of significance).

Despite the limitations of the study due to the small number of patients and the retrospective nature of the study, interesting new data have been identified that will allow better management of these patients in ART. First of all, before accepting these patients, the clinician will have to check with the referring doctors that there are no contraindications to pregnancy. For many authors (27–29), an FEV1 of less than 50% is predictive of a poor obstetrical and respiratory prognosis. Teratogenic treatments should be replaced. Regarding CFTR modulators, animal data are reassuring but human data are insufficient and caution is needed (30).

Jones et al. (31) noticed the occurrence of pregnancy in patients considered infertile after starting CFTR modulators with a reduction in conception time. Indeed, since these treatments improve the activity of the CFTR protein, we can suppose that these treatments have a direct positive impact on fertility and in particular on cervical mucus. Moreover, several women have also reported a normalization of their menstrual period on CFTR modulators. This observation supports the hypothesis of the involvement of the CFTR protein in ovarian function. Unfortunately, in this study, only one patient had received these treatments. It would be interesting in the area of these new therapies to evaluate in a future study the impact of these treatments on fertility parameters and on the prognosis in ART.

## Data Availability Statement

The original contributions presented in the study are included in the article/**Supplementary Material**. Further inquiries can be directed to the corresponding author.

## Ethics Statement

The studies involving human participants were reviewed and approved by CNIL. The patients/participants provided their written informed consent to participate in this study.

## Author Contributions

IB and NC designed the study, contributed to the discussions and manuscript. IB, AM, MP, ID, CR-J, and NC researched and interpreted data. SB, SH, and SL contributed to discussions and manuscript. All authors have approved the final version of the manuscript.

## Conflict of Interest

The authors declare that the research was conducted in the absence of any commercial or financial relationships that could be construed as a potential conflict of interest.

## Publisher’s Note

All claims expressed in this article are solely those of the authors and do not necessarily represent those of their affiliated organizations, or those of the publisher, the editors and the reviewers. Any product that may be evaluated in this article, or claim that may be made by its manufacturer, is not guaranteed or endorsed by the publisher.

## Supplementary Material

Supplementary Table 1 Fertility parameters of the population. Results are expressed as mean ± SD. Results are expressed as absolute values and as percentages in the group (column analysis). Normal cycles are defined by menstrual cycles of 25 to 30 days. Short cycles are defined by menstrual cycles of less than 25 days. Long cycles are defined by menstrual cycles of less than 30 days. NS, non-significant; AFC, antral follicle count; FSH, follicle stimulating hormone; PCT, postcoital test.

Supplementary Table 2 ART and pregnancy outcomes in CF women depending on ART procedures. Results are expressed as mean ± SD. Results are expressed as absolute values and as percentages for the outcome (column analysis). NS, non-significant; IVF, *in vitro* fertilisation; ICSI, intracytoplasmic sperm injection; IUI, intrauterine insemination; OI, ovulation induction; FET, frozen embryo transfer.

Supplementary Table 3 ART and pregnancy outcomes in control women depending on ART procedures. Results are expressed as mean ± SD. Results are expressed as absolute values and as percentages for the outcome (column analysis). NS, non-significant; IVF, *in vitro* fertilisation; ICSI, intracytoplasmic sperm injection; IUI, intrauterine insemination; OI, ovulation induction; FET, frozen embryo transfer.
