# Supplementary material for: The Prognosis of ART Is Not Altered in Cystic Fibrosis Women: A Case-Report Study
Source: Front Endocrinol (Lausanne). 2022 Feb 21;13:773753. doi: 10.3389/fendo.2022.773753 (PMC8898889; doi:10.3389/fendo.2022.773753)
Supplement: Supplementary Table 1 — Fertility parameters of the population. Results are expressed as mean ± SD. Results are expressed as absolute values and as percentages in the group (column analysis). Normal cycles are defined by menstrual cycles of 25 to 30 days. Short cycles are defined by menstrual cycles of less than 25 days. Long cycles are defined by menstrual cycles of less than 30 days. NS, non-significant; AFC, antral follicle count; FSH, follicle stimulating hormone; PCT, postcoital test. [file DataSheet_1.docx]

Table S1.

Fertility parameters of the population.

Results are expressed as mean ± SD.

Results are expressed as absolute values and as percentages in the group (column analysis).

Normal cycles are defined by menstrual cycles of 25 to 30 days. Short cycles are defined by menstrual cycles of less than 25 days. Long cycles are defined by menstrual cycles of less than 30 days.

NS: non significant.

AFC: antral follicle count.

FSH: follicle stimulating hormone.

PCT: postcoital test.

|  | CF  (n= 17) | Controls  (n= 34) | p |
| --- | --- | --- | --- |
| AFC > 5 | 15 (93.8%) | 32 (100%) | NS |
| AFC < 5 | 1 (6.2%) | 0 (0%) |  |
| Missing data | 1 | 2 |  |
| AFC mean | 19.5 (± 10.8) | 26.8 (± 13.5) | NS |
| FSH < 2 UI/L | 0 (0%) | 1 (3%) | NS |
| FSH > 10 UI/L | 1 (7.1%) | 1 (3%) |  |
| FSH= 2 to 10 UI/L | 13 (92.9%) | 31 (93.9%) |  |
| Missing data | 3 | 1 |  |
| FSH mean (UI/L) | 6.3 (± 2.2) | 6.9 (± 1.8) | NS |
| PCT negative | 9 (100%) | 7 (77.8%) | NS |
| PCT positive | 0 (0%) | 2 (22.2%) |  |
| Missing data | 8 | 25 |  |
| No tubal disorder | 10 (76.9%) | 27 (87.1%) | NS |
| Tubal disorder | 3 (23.1%) | 4 (12.9%) |  |
| Missing data | 4 | 3 |  |
| Spontaneous menarche after 15 yrs | 3 (23.1%) | 3 (30%) | NS |
| Spontaneous menarche before 15 yrs | 10 (76.9%) | 7 (70%) |  |
| Missing data | 4 | 25 |  |
| Menarche age (yrs) | 13.8 (± 1.5) | 11.8 (± 4.2) | NS |
| Normal cycles | 13 (76.5%) | 19 (55.9%) | NS |
| Short cycles | 0 (0%) | 4 (11.8%) |  |
| Long cycles | 4 (23.5%) | 11 (32.4%) |  |

Table S2.

ART and pregnancy outcomes in CF women depending on ART procedures.

Results are expressed as mean ± SD.

Results are expressed as absolute values and as percentages for the outcome (column analysis).

NS: non significant.

IVF: in vitro fertilisation.

ICSI: intracytoplasmic sperm injection.

IUI: intrauterine insemination.

OI: ovulation induction.

FET: frozen embryo transfer.

|  | IVF/ICSI  (n= 18) | IUI  (n= 26) | OI  (n= 3) | FET  (n= 19) | p |
| --- | --- | --- | --- | --- | --- |
| Ovarian hyperstimulation | 3 (16.7%) | 0 (0%) | 0 (0%) | 0 (0%) | 0.0432 |
| No ovarian hyperstimulation | 15 (83.3%) | 26 (100%) | 3 (100%) | 19 (100%) |  |
| HCG > 100 UI/L | 7 (38.9%) | 7 (26.9%) | 0 (0%) | 5 (26.3%) | NS |
| HCG < 100 UI/L | 2 (11.1%) | 0 (0%) | 0 (0%) | 1 (5.3%) |  |
| HCG negative | 9 (50.0%) | 19 (73.1%) | 3 (100%) | 13 (68.4%) |  |
| Clinical pregnancy | 6 (33.3%) | 7 (26.9%) | 0 (0%) | 4 (21.1%) | 0.61  NS |
| No clinical pregnancy | 12 (66.7%) | 19 (73.1%) | 3 (100%) | 15 (78.9%) |  |
| Pregnancy loss, ectopic pregnancy | 4 (22.2%) | 1 (3.8%) | 0 (0%) | 3 (15.8%) | NS |
| No pregnancy loss or ectopic pregnancy | 14 (77.8%) | 25 (96.2%) | 3 (100%) | 16 (84.2%) |  |
| Live births | 3 (16.7%) | 6 (23.1%) | 0 (0%) | 1 (5.3%) | NS |
| No live births | 15 (83.3%) | 20 (76.9%) | 3 (100%) | 18 (94.7%) |  |

Table S3.

ART and pregnancy outcomes in control women depending on ART procedures.

Results are expressed as mean ± SD.

Results are expressed as absolute values and as percentages for the outcome (column analysis).

NS: non significant.

IVF: in vitro fertilisation.

ICSI: intracytoplasmic sperm injection.

IUI: intrauterine insemination.

OI: ovulation induction.

FET: frozen embryo transfer.

|  | IVF/ICSI (n= 64) | IUI  (n= 44) | OI  (n= 17) | FET  (n= 30) | p |
| --- | --- | --- | --- | --- | --- |
| Ovarian hyperstimulation | 10 (15.6%) | 0 (0%) | 0 (0%) | 0 (0%) | 0.0018 |
| No ovarian hyperstimulation | 54 (84.4%) | 44 (100%) | 17 (100%) | 30 (100%) |  |
| HCG > 100 UI/L | 17 (26.6%) | 5 (11.3%) | 1 (5.9%) | 12 (40.0%) | 0.0011 |
| HCG < 100 UI/L | 0 (0%) | 1 (2.3%) | 0 (0%) | 3 (10.0%) |  |
| HCG negative | 47 (73.4%) | 38 (86.4%) | 16 (94.1%) | 15 (50.0%) |  |
| Clinical pregnancy | 16 (25.0%) | 5 (11.4%) | 1 (5.9%) | 12 (40.0%) | 0.009 |
| No clinical pregnancy | 48 (75.0%) | 39 (88.6%) | 16 (94.1%) | 18 (60.0%) |  |
| Pregnancy loss, ectopic pregnancy | 4 (6.2%) | 0 (0%) | 0 (0%) | 8 (26.7%) | 4e-04 |
| No pregnancy loss or ectopic pregnancy | 60 (93.8%) | 44 (100%) | 17 (100%) | 22 (73.3%) |  |
| Live births | 13 (20.3%) | 5 (11.4%) | 1 (5.9%) | 4 (13.3%) | NS |
| No live births | 51 (79.7%) | 39 (88.6%) | 16 (94.1%) | 26 (86.7%) |  |
